# Integrated Metabolomics and Transcriptomics Provided Novel Insights into the Biosynthetic Regulation of Phenolic Compounds in *Vitis heyneana* Roem. et Schult. var. *adenoclada* (Hand.-Mazz.)

**DOI:** 10.3390/foods15142574

**Published:** 2026-07-22

**Authors:** Yulu Miao, Zhaofei Lan, Rongfu Wei, Jinbiao Liu, Yingfen Yu, Jin Zhang, Mengna Huang, Yibin Lan, Yongmei Zhou, Fengping Pan, Haifeng Jia, Guo Cheng, Sihong Zhou

**Affiliations:** 1 College of Agriculture, Guangxi University, Nanning 530004, China; 2 Grape and Wine Research Institute, Guangxi Academy of Agricultural Sciences, Nanning 530007, China; 3 Center for Viticulture and Enology, College of Food Science & Nutritional Engineering, China Agricultural University, Beijing 100083, China

**Keywords:** *Vitis heyneana* var. *adenoclada*, metabolomics, transcriptomics, phenolic compounds, phenylpropanoid pathway, MYB transcription factors

## Abstract

*Vitis heyneana* Roem. et Schult. var. *adenoclada* (Hand.-Mazz.) is an important wild grapevine resource with unique berry traits and wine quality characteristics; yet the molecular mechanisms governing its phenolic biosynthesis remain to be further elucidated compared with cultivated *Vitis vinifera*. This study aimed to systematically characterize the transcriptional and metabolic regulation of phenolic compound accumulation in *V. heyneana* var. *adenoclada* using integrated metabolomics and transcriptomics. At the metabolic level, *V. heyneana* var. *adenoclada* exhibited significantly higher contents of phenolic acids (caffeic and ferulic acids), resveratrol, flavonols (quercetin and myricetin), flavan-3-ols (catechin and epicatechin), and anthocyanin diglucosides compared with *V. vinifera* cv. Cabernet Sauvignon. The two tested *V. heyneana* varieties showed distinct developmental accumulation profiles: Guiheizhenzhu No. 6 accumulated more ferulic acid, resveratrol, myricetin, and anthocyanins at maturity, whereas Yeniang No. 2 showed higher quercetin and flavan-3-ols at the green stage. Integrated multi-omics correlation analyses revealed that the elevated phenolic content is driven by the coordinated upregulation of key structural genes mediated by a complex MYB regulatory network. Specifically, *MYB12* (*MYBF1*) positively correlated with *COMT*, *CHI*, *DFR*, and *LAR*. *MYB5*, *MYBS3*, and *MYB61* promoted quercetin synthesis by activating *FLS*. *MYB06*, *MYB4*, and *MYB36* enhanced *STS* transcription to drive resveratrol biosynthesis, whereas *MYB1* and *MYB5* cooperatively regulated *UFGT*, *5GT*, and *GST4*, facilitating efficient synthesis and transport of anthocyanin diglucosides. These findings provide comprehensive mechanistic insights into phenolic metabolism in *V. heyneana* var. *adenoclada* and establish a valuable theoretical foundation for improving the flavor quality and regional utilization of wild grape wines.

## 1. Introduction

*Vitis heyneana* Roem. et Schult. var. *adenoclada* (Hand.-Mazz.), a wild grapevine species endemic to China, is widely distributed in the southern regions of the Yangtze River, including Hunan, Fujian, and Guangxi Provinces. Taxonomically, it belongs to East Asia within the section *Vitis* [1]. This species is characterized by three sexual types based on floral organ morphology: hermaphroditic, female, and male. Its most distinctive morphological trait is the dense covering of glandular hairs on various plant parts, such as young leaves, new shoots, and inflorescences, which serves as a key diagnostic feature distinguishing it from closely related species [2,3,4]. *V. heyneana* var. *adenoclada* exhibits strong environmental adaptability and disease resistance, with low susceptibility to anthracnose, powdery mildew, and downy mildew even under the highly humid conditions of southern China [5]. Moreover, it exhibits superior tolerance to waterlogging, heat, and drought and shows higher yield and photosynthetic efficiency compared to cultivated varieties, positioning it as a valuable genetic resource for breeding programs aimed at enhancing photosynthesis and stress resilience [6].

Phenolic compounds are major contributors to grape and wine quality, playing essential roles in determining wine color, astringency, bitterness, and aging potential, while also contributing to sensory complexity and nutritional value [7]. Phenolic compounds mainly include phenolic acids (e.g., caffeic and ferulic acids), flavan-3-ols (e.g., catechin and epicatechin), flavonols (e.g., quercetin and myricetin), stilbenes (e.g., resveratrol), and anthocyanins [8]. Phenolic biosynthesis primarily occurs through the phenylpropanoid pathway, beginning from the aromatic amino acids phenylalanine and tyrosine. Through the catalytic actions of key enzymes such as phenylalanine ammonia-lyase (PAL), cinnamate 4-hydroxylase (C4H), and 4-coumarate-CoA ligase (4CL), 4-coumaroyl-CoA is generated, which subsequently enters various branch pathways to produce diverse phenolic compounds [9]. This metabolic pathway is regulated by a complex network encompassing multiple structural genes, which encode key biosynthetic enzymes such as caffeic acid 3-*O*-methyltransferase (COMT), chalcone synthase (CHS), stilbene synthase (STS), and UDP-glucose: flavonoid 3-*O*-glucosyltransferase (UFGT). Additionally, the network involves various transcription factors (TFs), including MYB, basic/helix-loop-helix (bHLH), and WRKY families [10,11,12]. Studies have shown that MYB TFs play a key role in regulating phenolic biosynthesis, often forming MBW complexes with bHLH and WD40 proteins to activate the expression of downstream structural genes [13].

The accumulation of phenolic compounds in grapes is influenced by a complex interplay of factors, including genetic background, cultivation practices, and climatic conditions. Among these factors, genetic background is a key determinant of the composition and content of phenolic compounds. Extensive studies have shown that East Asian *Vitis* species are characterized by high accumulation of anthocyanin-3,5-*O*-diglucosides, abundant flavan-3-ols and phenolic acids, and a notably high resveratrol content in some genotypes, particularly under stress conditions [14,15,16]. In a previous study, we performed whole-genome sequencing of *V. heyneana* var. *adenoclada* Guiheizhenzhu No. 6 (GH6) [17], laying a genetic foundation for further studies. Subsequent studies focused on the qualitative characteristics of different *V. heyneana* var. *adenoclada* cultivars. For instance, Guiheizhenzhu No. 3 from Luocheng contains higher levels of resveratrol, tannins, and characteristic aroma compounds compared to that from Nanning [18]. Geographical origin has been identified as the most significant factor affecting the physicochemical indices and flavonoid metabolic profiles of Yeniang No. 2 (YN2) berries [19]. Furthermore, semi-shading has been shown to enhance the contents of sugars, most organic acids, and anthocyanins in Guiheizhenzhu No. 4 [20]. Comparative studies on the stability of anthocyanins under varying temperature and pH conditions have revealed differences between GH6 or YN2 and *V. vinifera* Cabernet Sauvignon (CS) [21]. Despite these findings, the accumulation dynamics of phenolic compounds in *V. heyneana* var. *adenoclada* across developmental stages and the underlying molecular regulatory mechanisms remain unclear.

In recent years, the rapid advancement of metabolomics and transcriptomics technologies has provided powerful tools for elucidating complex biosynthetic pathways in non-model plant species [22,23]. Metabolomics, as an important branch of systems biology, has been recognized as a “biochemical readout” that bridges the genotype and phenotype [24,25]; its high-throughput analytical platforms (e.g., LC-MS/MS) enable the simultaneous detection and quantification of hundreds to thousands of metabolites in plants [26]. Metabolomics allows comprehensive profiling of metabolites, while transcriptomics permits global analysis of gene expression; the integration of these two approaches has proven particularly effective in revealing the regulatory networks underlying secondary metabolism in various plant species [24,27,28].

This study investigated the accumulation patterns and regulatory mechanisms of phenolic compounds in *V. heyneana* var. *adenoclada* GH6 and YN2, with *V. vinifera* CS used as a control. Integrated metabolomic and transcriptomic analyses were performed using ultra-performance liquid chromatography–tandem mass spectrometry (UPLC–MS/MS) and RNA sequencing (RNA-seq). The objectives of this study were to identify key phenolic compounds and their dynamic changes, determine key structural genes and regulators involved in phenolic metabolism, and construct molecular regulatory networks underlying phenolic biosynthesis. This study not only addresses the existing knowledge gap in systematic phenolic profiling and molecular regulation of wild grape resources but also provides a crucial theoretical foundation for improving grape quality and developing functional foods using unique genetic resources from grapes.

## 2. Materials and Methods

### 2.1. Experimental Materials

Grape berry samples were collected from the Mingyang Double-Cropping Grape Experimental Base of the Guangxi Academy of Agricultural Sciences (22°36′34″ N, 108°14′33″ E). The experimental materials consisted of two *V. heyneana* var. *adenoclada* cultivars, namely, Guiheizhenzhu No. 6 (GH6) and Yeniang No. 2 (YN2), with *V. vinifera* Cabernet Sauvignon (CS) used as a control. The vineyard was established on yellow loam soil with a plant spacing of 1.8 m × 3.0 m and north–south row orientation. The vines exhibited moderate vigor and were managed using conventional practices for fertilization, irrigation, and pest control. Samples were collected during the 2023 growing season at four key developmental stages according to the E–L system [29]: berries still hard and green (E–L 33), berries begin to colour and enlarge (E–L 35), berries not quite ripe (E–L 37), and berries harvest-ripe (E–L 38). Nine uniformly growing vines were selected as sample sources, with every three vines constituting one biological replicate. From each biological replicate, a minimum of 300 berries were randomly collected from at least 30 clusters. All samples were immediately placed in ice boxes for transport to the laboratory. For each biological replicate, 30 berries were used for basic physicochemical analysis. The remaining berries were snap-frozen in liquid nitrogen and stored at −80 °C for subsequent metabolomic and transcriptomic analyses.

### 2.2. Analysis of Basic Physicochemical Parameters

The physicochemical properties of grape berries were evaluated using standardized methods. The average berry weight was determined by weighing three sets of 30 intact berries randomly collected from each biological replicate using an analytical balance (ME204E, Mettler Toledo, Greifensee, Switzerland). Soluble solid content (SSC) was measured using a portable digital refractometer (PAL-1, Atago Co., Ltd., Tokyo, Japan) and expressed as °Brix. The pH was measured using a laboratory pH meter (PHS-3C, INESA Scientific Instrument Co., Ltd., Shanghai, China). Titratable acidity (TA) was measured through sodium hydroxide neutralization titration and expressed as tartaric acid equivalents in accordance with standard OIV analytical guidelines [30].

### 2.3. Metabolite Extraction and Analysis

#### 2.3.1. Sample Preparation and Extraction

All biological samples were freeze-dried using a lyophilizer (Scientz-100F, Ningbo Scientz Biotechnology Co., Ltd., Ningbo, China) and then ground to powder using a ball mill (MM 400, Retsch GmbH, Haan, Germany) at 30 Hz for 1.5 min. Subsequently, 50 mg of the powdered sample was accurately weighed and mixed with 1200 μL of pre-cooled (−20 °C) 70% aqueous methanol containing internal standards. The mixture was vortexed for 30 s at 30 min intervals (six times in total). After centrifugation at 12,000× *g* for 3 min, the supernatant was filtered through a 0.22 μm microporous membrane and stored in vials for UPLC-MS/MS analysis.

#### 2.3.2. UPLC-MS/MS Conditions

The sample extracts were analyzed using a UPLC-ESI-MS/MS system (ExionLC™ AD, SCIEX, Framingham, MA, USA) coupled with a QTRAP mass spectrometer. Chromatographic separation was performed on an Agilent SB-C18 column (1.8 µm, 2.1 mm × 100 mm, Agilent Technologies, Santa Clara, CA, USA) maintained at 40 °C. The mobile phase consisted of (A) water with 0.1% formic acid and (B) acetonitrile with 0.1% formic acid, with the following gradient program: 0–9 min, linear gradient from 95% A to 5% A; 9–10 min, 5% A; 10–11.1 min, linear gradient to 95% A; and 11.1–14 min, 95% A. The flow rate was 0.35 mL/min, and the injection volume was 2 μL.

#### 2.3.3. ESI-QTRAP-MS/MS Parameters

Mass spectrometric detection was performed using a QTRAP mass spectrometer (SCIEX, Framingham, MA, USA) with an ESI source. The ESI source was operated at 550 °C with ion spray voltages of 5500 V (positive mode) and −4500 V (negative mode). The ion source gas I (GSI), gas II (GSII), and curtain gas (CUR) were set at 50, 60, and 25 psi, respectively, and collision-activated dissociation (CAD) was set to high. Data acquisition was performed in multiple reaction monitoring (MRM) mode with collision gas (nitrogen) set to medium. Declustering potential (DP) and collision energy (CE) for individual MRM transitions were optimized for each analyte.

#### 2.3.4. Metabolomic and Statistical Data Analysis

Metabolomics analysis was conducted following the standard procedures of MetWare Biotechnology Co., Ltd. (Wuhan, China) [31]. Raw data were processed using Analyst 1.6.3 (SCIEX, Framingham, MA, USA). Metabolites were identified against the MetWare database based on retention time, mass error (≤5 ppm), and match factor (≥0.7), and annotated using the Kyoto Encyclopedia of Genes and Genomes (KEGG) database [32]. Pathway enrichment analysis was conducted via Metabolite Sets Enrichment Analysis (MSEA) based on the KEGG Pathway database, with significance determined by hypergeometric test *p*-values.

Unsupervised principal component analysis (PCA) was performed using the prcomp function in R (version 4.5.1) with unit variance scaling [33]. The first two principal components (PC1 and PC2) were extracted to visualize the overall metabolic variance, with eigenvalues > 1.0 considered significant. Hierarchical cluster analysis (HCA) and Pearson correlation coefficients between samples were carried out using the R (version 4.5.1) package ComplexHeatmap (version 2.18.0) [34], with Euclidean distance and Ward’s linkage method. For differential metabolite selection, orthogonal partial least squares discriminant analysis (OPLS-DA) was performed with log2-transformed and mean-centered data [35]. Metabolites with variable importance in projection (VIP) > 1.0 and |Log2FC| ≥ 1.0 were considered significantly different. Metabolites with Log2FC ≥ 1.0 and VIP > 1.0 were defined as up-regulated (increased accumulation), and those with Log2FC ≤ −1.0 and VIP > 1.0 were defined as down-regulated (decreased accumulation). A permutation test with 200 iterations was conducted to validate the OPLS-DA model and prevent overfitting.

### 2.4. Transcriptomic Sequencing and Analysis

#### 2.4.1. RNA Extraction, Library Construction, and Sequencing

Total RNA was extracted from grape berry samples using the CTAB-PBIOZOL method with ethanol precipitation [36]. RNA integrity, concentration, and purity were assessed using a Qubit fluorometer (Qubit 4.0, Thermo Fisher Scientific, Waltham, MA, USA) and a Qsep400 high-throughput biofragment analyzer (Qsep400, BiOptic Inc., New Taipei City, Taiwan, China); samples with RNA integrity number (RIN) ≥ 7.0 were used for subsequent library construction.

mRNA was enriched from total RNA using Oligo(dT)-coated magnetic beads, fragmented, and reverse-transcribed into cDNA using random hexamer primers. For strand-specific library construction, dUTPs were incorporated during second-strand cDNA synthesis. The libraries were subjected to end repair, A-tailing, adapter ligation, and PCR amplification. Library quality and concentration were assessed using a Qubit fluorometer and Qsep400 analyzer, and the effective concentration was quantified by qPCR. Qualified libraries were sequenced on the Illumina platform (NovaSeq 6000, Illumina, San Diego, CA, USA) to generate 150 bp paired-end reads.

#### 2.4.2. Quality Control and Sequence Alignment

Raw reads were preprocessed using fastp (version 0.20.0) to remove adapter sequences and low-quality reads (reads with ambiguous ‘N’ bases exceeding 10% or with low-quality bases (Q ≤ 20) exceeding 50% of the read length). The obtained clean reads were then aligned to the chromosome-level high-quality reference genome of *V. heyneana* var. *adenoclada* Guiheizhenzhu No.6 (GH6) (NGDC BioProject: PRJCA009537) using HISAT2 (version 2.2.1) [17]. The reference genome assembly is publicly accessible via the NGDC official website (https://ngdc.cncb.ac.cn).

#### 2.4.3. Transcript Assembly and Quantification

Transcript assembly was performed using StringTie (version 2.1.0). Gene expression levels were quantified using featureCounts (version 2.0.1), and Fragments Per Kilobase of transcript per Million mapped reads (FPKM) values were calculated for each gene.

#### 2.4.4. Differential Expression and Enrichment Analysis

Differential expression analysis between groups was performed using DESeq2 (version 1.30.1). *p*-values were adjusted using the Benjamini & Hochberg method, and genes with |Log2FC| ≥ 1 and FDR < 0.05 were considered significantly differentially expressed. Functional enrichment analysis of differentially expressed genes was conducted using KEGG pathway enrichment analysis based on the hypergeometric distribution test.

### 2.5. Transcription Factor (TF) Analysis

Transcription factors (TFs) were predicted based on the transcriptomic data using the Plant TFDB database (https://planttfdb.gao-lab.org/). Pearson’s correlation coefficients (PCCs) were calculated to assess the relationships between genes and TFs. Interaction networks were visualized using Cytoscape (version 3.10.2).

### 2.6. Weighted Gene Co-Expression Network Analysis

Weighted gene co-expression network analysis (WGCNA) was performed using the WGCNA R package (version 1.68) [37] to construct co-expression networks from DEGs and correlate them with phenolic metabolite traits. The analysis was based on a scale-free topology criterion. An adjacency matrix was constructed and transformed into a topological overlap matrix, followed by hierarchical clustering to identify co-expression modules. Key parameters included a soft-thresholding power of 10, minimum module size of 30, and module merging threshold of 0.5.

### 2.7. Statistical Analysis

All statistical calculations were performed using Microsoft Excel 2019 (Microsoft Corp., Redmond, WA, USA). One-way analysis of variance (ANOVA) was conducted on the Metware Cloud platform (https://cloud.metware.cn) to assess intergroup differences, followed by Duncan’s new multiple range test for post hoc comparisons, with statistical significance set at *p* < 0.05. All experiments were performed in triplicate, and data are presented as mean ± standard deviation (SD) (*n* = 3).

## 3. Results

### 3.1. Analysis of Physicochemical Properties at Harvest

As shown in Table 1, basic physicochemical characteristics varied among the three grape cultivars at harvest. No significant difference was observed in single berry weight among the three cultivars. SSC followed the order of CS > GH6 > YN2, with *V. heyneana* var. *adenoclada* showing a significantly lower SSC than *V. vinifera* CS. The pH of CS was significantly higher than that of GH6 and YN2. TA did not show significant differences between the two *V. heyneana* var. *adenoclada* cultivars, with YN2 and GH6 exhibiting TA values of 17.23 and 16.9 g/L, respectively, both significantly higher than that of CS.

### 3.2. Overview of Metabolite Profiles

**Figure 1:**
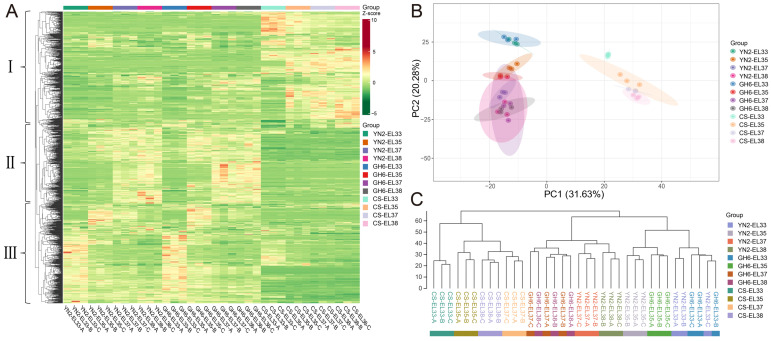
Metabolomic profiling of three grape varieties across four developmental stages (E–L system): (**A**) overview of the 1281 metabolites identified from samples containing biological replicates, where Roman numerals (I–III) indicate major metabolite clusters, and the color scale represents Z-score values ranging from −5 to 10; (**B**) principal component analysis (PCA) of metabolite profiles, with shaded areas representing 95% confidence ellipses for each group; (**C**) clustering dendrogram based on metabolomic data.

A comparative analysis of metabolite profiles revealed differences among the three cultivars at different developmental stages (Figure 1A). Hierarchical clustering categorized the identified metabolites into three clusters: cluster I metabolites were preferentially accumulated in CS, cluster II metabolites showed a higher abundance in GH6 and YN2 at maturity, and cluster III metabolites were predominantly enriched in GH6 and YN2 at E–L 33 and E–L 35. PCA separated *V. heyneana* var. *adenoclada* from *V. vinifera* CS, with biological replicates clustered together tightly (Figure 1B). The first principal component (PC1), explaining 31.06% of the total variance, effectively distinguished GH6 and YN2 from CS. The second principal component (PC2) further separated samples based on three developmental stages: berries still hard and green, onset of veraison, and harvest stage. These results were consistent with the hierarchical clustering dendrogram (Figure 1C).

**Figure 2:**
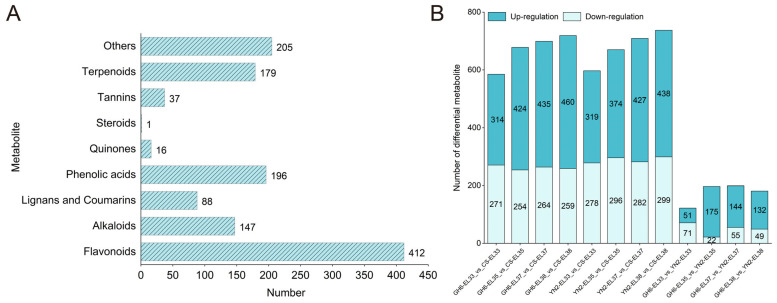
Classification and comparison of metabolites in grape berries of three cultivars: (**A**) statistics of the identified and classified metabolites across all biological samples; (**B**) numbers of differentially accumulated metabolites (DAMs) between pairwise comparisons at different developmental stages (E–L system). Dark blue and light blue segments represent up- and down-regulated metabolites, respectively (|Log2FC| ≥ 1), with total numbers shown above each bar.

Overall, 1281 metabolites were identified using UPLC–MS/MS analysis and a self-constructed database (Table S1). These metabolites were categorized into flavonoids (412), phenolic acids (196), terpenoids (179), alkaloids (147), lignans and coumarins (88), tannins (37), quinones (16), steroids (1), and others (205), with flavonoids being the most abundant class (Figure 2A). To examine the effects of cultivar and developmental stage on metabolite composition, pairwise comparisons of differentially accumulated metabolites (DAMs) were performed among the three grape subjects across their respective E–L developmental stages (Figure 2B). The number of upregulated metabolites significantly exceeded that of downregulated metabolites in a majority of the comparisons. Notably, the number of differential metabolites between cultivars was substantially higher than that between developmental stages. Specifically, the fewest differential metabolites were observed between GH6 and YN2, whereas similar numbers were detected for GH6 versus CS and YN2 versus CS. The number of differential metabolites exhibited a consistent increase throughout fruit development. In comparisons between *V. heyneana* var. *adenoclada* and *V. vinifera* CS, the lowest count was observed at E–L 33, whereas the highest count was observed at E–L 38.

The number of differential metabolites between *V. heyneana* var. *adenoclada* and *V. vinifera* CS peaked at E–L 38, indicating the most pronounced difference in metabolite profiles at this stage. To elucidate the underlying regulatory mechanisms, KEGG pathway enrichment analysis was performed on the differential metabolites identified between GH6-EL 38 and CS-EL 38 and between YN2-EL 38 and CS-EL 38 (Figure S1). The results revealed significant enrichment of the metabolites in several key pathways, including phenylpropanoid biosynthesis, phenylalanine metabolism, flavone and flavonol biosynthesis, anthocyanin biosynthesis, and isoflavonoid biosynthesis. These findings collectively highlight the key role of phenolic biosynthesis in shaping the metabolic profiles of the three cultivars.

### 3.3. Differential Accumulation of Phenolic Compounds Among Grape Cultivars

**Figure 3:**
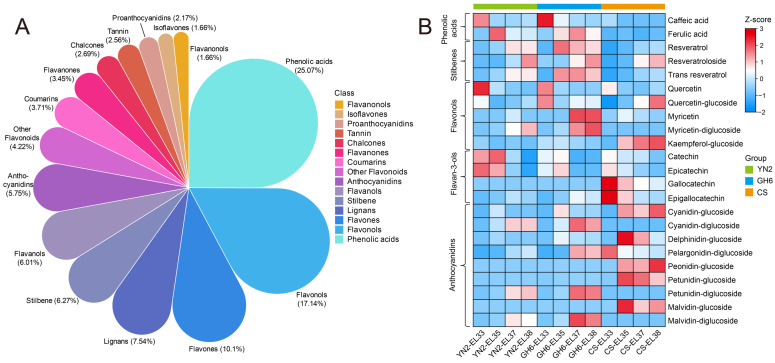
Profiling of phenolic metabolites across three grape varieties: (**A**) classification of phenolic metabolites across all samples; (**B**) heatmap of phenolic compound profiles among varieties, with blue-to-red color scale from low to high abundance.

To assess the metabolite accumulation patterns of *V. heyneana* var. *adenoclada*, K-means clustering analysis was performed, categorizing metabolites across the three cultivars into seven subclasses based on their expression profiles (Figure S2). At maturity, 218 metabolites from subclass 7 exhibited a higher abundance in *V. heyneana* var. *adenoclada* than in *V. vinifera*. At E–L 33 and E–L 35, subclasses 1 and 5 contained 241 and 212 metabolites, respectively, with these metabolites being more abundant in *V. heyneana* var. *adenoclada* (Table S2). In terms of metabolite composition, flavonoids (48.17%) and phenolic acids (15.14%) predominated in subclass 7, with these categories also showing substantial proportions in subclasses 1 and 5 (Figure S3). A systematic analysis of phenolic compounds across the three cultivars revealed 782 phenolic metabolites, with phenolic acids being the most abundant category (25.07%), followed by flavonols (Figure 3A).

As shown in Figure 3B, phenolic acids were more abundant in *V. heyneana* var. *adenoclada* than in *V. vinifera*, with their accumulation patterns exhibiting genotype- and developmental stage-specificity. In particular, caffeic acid showed higher accumulation in GH6 and YN2 at the green berry stage, with GH6 exhibiting a higher caffeic acid content than YN2. Conversely, ferulic acid content increased as the berries matured. Among stilbenes, both resveratrol and its glycoside (resveratroloside) showed markedly higher levels at maturity, with GH6 showing the highest levels, followed by YN2 and CS. Among flavonols, quercetin was more abundant at the green berry stage than at maturity, with its content following the order of YN2 > GH6 > CS. Myricetin levels were higher in GH6 during late maturity, whereas kaempferol was the most abundant in CS at maturity. Among flavan-3-ols, catechin and epicatechin showed gradually decreasing contents during berry development, with YN2 exhibiting higher levels of both compounds at the green berry and veraison stages. Gallocatechin and epigallocatechin were the most abundant in CS at the green berry stage; however, their levels decreased as maturation progressed. Anthocyanin accumulation also showed significant developmental stage-specific trends. Anthocyanin-3-*O*-glucoside levels sharply increased in CS after entry into the maturation phase, whereas anthocyanin-3,5-*O*-diglucosides were more abundant in mature GH6 and YN2 berries, with higher levels in GH6 than in YN2.

### 3.4. Transcriptomic Sequencing and Quality Assessment

After alignment to the reference genome, approximately 46.45–58.43 million high-quality reads were obtained from each library (Table S3). The alignment rates ranged from 88.88% to 98.03%. Quality assessment revealed a Q30 score of 93.99–94.98%, and GC content was uniformly distributed across samples, ranging from 46.11% to 47.23%. These metrics collectively suggested that the sequencing data were highly reliable, exhibited stable quality, and showed no significant base composition bias, confirming their suitability for subsequent in-depth analysis.

**Figure 4:**
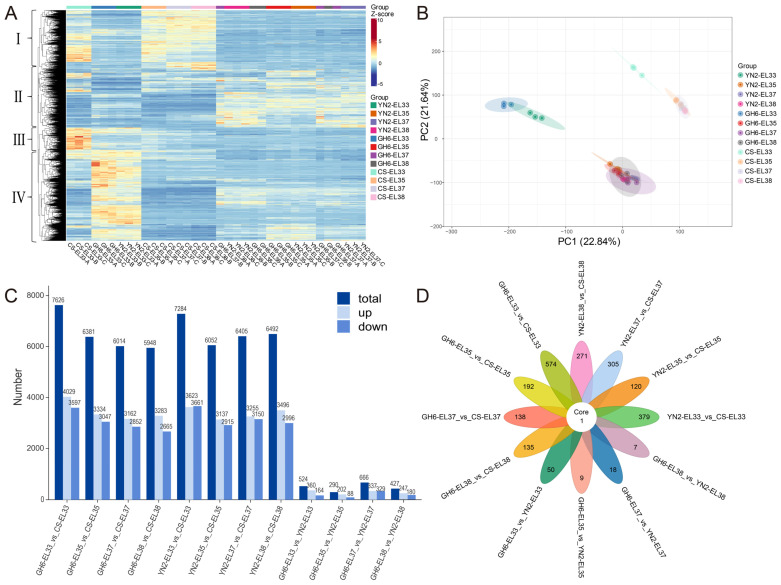
Transcriptomic profiling of three grape varieties across developmental stages (E–L system): (**A**) overview of biological replicates for the 13,444 DEGs identified from 36 samples (with biological replicates), where Roman numerals (I–IV) indicate major expression clusters, and the color scale represents Z-score values ranging from −5 to 10; (**B**) PCA of transcriptomic data, with shaded areas representing 95% confidence ellipses for each group; (**C**) numbers of DEGs among the three varieties at different developmental stages; (**D**) Venn diagram showing shared and unique DEGs among the three varieties.

Transcriptomic analysis of 36 grape samples revealed the expression of 48,912 genes, including 1393 novel genes. Based on the screening thresholds of |log2FC| ≥ 1 and FDR < 0.05, 13,444 DEGs were identified (Table S4). Based on their expression patterns across the three grape cultivars, these DEGs were categorized into four clusters (Figure 4A). Cluster I contained genes highly expressed in CS, cluster II contained genes upregulated in *V. heyneana* var. *adenoclada* after veraison, cluster III contained genes predominantly expressed in CS during the green berry stage, and cluster IV contained genes highly expressed in *V. heyneana* var. *adenoclada* during the green berry stage. The PCA results were consistent with this clustering, further validating the reliability of the observed intergroup differences (Figure 4B).

As shown in Figure 4C, a comparative analysis of gene expression profiles among the three grape cultivars revealed substantial transcriptomic differences between *V. heyneana* var. *adenoclada* and *V. vinifera*, with the most pronounced difference observed at E–L 33. Specifically, 7626 DEGs were identified between GH6-EL 33 and CS-EL 33, whereas 7284 DEGs were identified between YN2-EL 33 and CS-EL 33. Although the number of DEGs decreased during fruit development, it remained consistently high throughout the process. Notably, the number of DEGs was substantially smaller between GH6 and YN2 than between either of these lines and CS, with a minimum count observed at E–L 35 and a relative peak observed at E–L 37. Venn diagram analysis indicated that multiple genes were differentially expressed during various stages, with only one gene being consistently differentially expressed throughout all stages (Figure 4D). These results establish significant divergence in gene expression regulatory mechanisms between *V. heyneana* var. *adenoclada* and *V. vinifera*. Although GH6 and YN2 exhibit considerably similar transcriptomic profiles at specific stages, potential differentiation in key regulatory pathways may occur at E–L 37.

**Figure 5:**
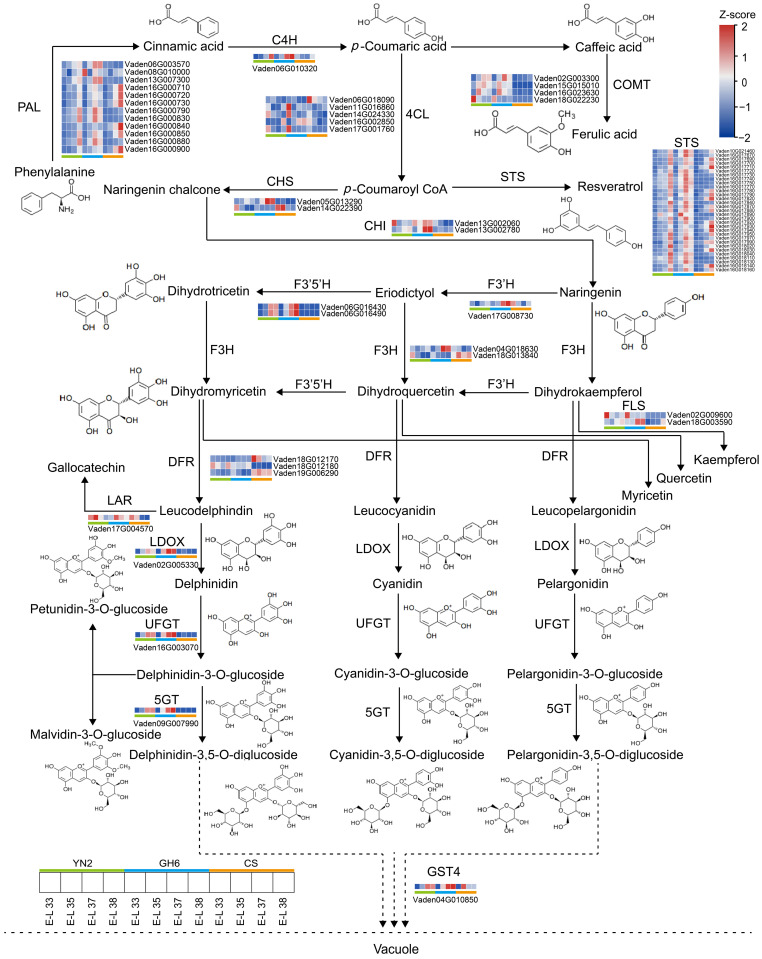
Expression patterns of key structural genes involved in phenolic compound biosynthesis in three grape varieties at different developmental stages (E–L system). The heatmap color scale from blue to red represents low to high expression levels (log2-transformed FPKM values). Structural genes encoding key enzymes in the phenylpropanoid and flavonoid pathways are shown: PAL, phenylalanine ammonia-lyase; C4H, cinnamate 4-hydroxylase; 4CL, 4-coumarate-CoA ligase; COMT, caffeic acid *O*-methyltransferase; CHS, chalcone synthase; CHI, chalcone isomerase; STS, stilbene synthase; F3′H, flavonoid 3′-hydroxylase; F3′5′H, flavonoid 3′,5′-hydroxylase; FLS, flavonol synthase; DFR, dihydroflavonol 4-reductase; LAR, leucoanthocyanidin reductase; LDOX, leucoanthocyanidin dioxygenase; UFGT, UDP-glucose:flavonoid 3-*O*-glucosyltransferase; 5GT, anthocyanin 5-*O*-glucosyltransferase; GST4, glutathione S-transferase 4.

The gene expression dynamics of structural genes involved in phenylpropanoid and flavonoid/stilbene biosynthesis were assessed across the three grape cultivars using RNA-seq. The results are presented in Figure 5. RNA-seq revealed different expression patterns of genes encoding key enzymes related to phenolic biosynthesis and transport among the three cultivars. The expression levels of the caffeic acid *O*-methyltransferase gene (*COMT*) and cinnamate 4-hydroxylase gene (*C4H*) were significantly higher in *V. heyneana* var. *adenoclada* than in CS; however, these genes showed opposite expression patterns between YN2 and GH6. In particular, *COMT* expression was higher in YN2, whereas *C4H* expression was higher in GH6. Phenylalanine ammonia-lyase gene (*PAL*) and stilbene synthase gene (*STS*) exhibited highly consistent expression profiles, with their transcript levels markedly increasing at maturity in all three cultivars. Conversely, 4-coumarate-CoA ligase gene (*4CL*) showed the highest expression during the green berry stage in all cultivars. Chalcone isomerase gene (*CHI*), flavonoid 3′-hydroxylase gene (*F3′H*), and flavonoid 3′,5′-hydroxylase gene (*F3′5′H*) showed similar expression trends across cultivars and developmental stages. These genes were more highly expressed in *V. heyneana* var. *adenoclada* than in CS. All these genes showed peak expression levels in GH6 at maturity, with the levels significantly exceeding those observed in the other two cultivars at the same stage or in the same cultivar at other stages. Both flavonol synthase gene (*FLS*) and leucoanthocyanidin reductase gene (*LAR*) maintained relatively high expression levels in *V. heyneana* var. *adenoclada*, with inter-cultivar differences being more pronounced than variations across developmental stages within the same cultivar. Leucoanthocyanidin dioxygenase gene (*LDOX*), UDP-glucose: flavonoid 3-*O*-glucosyltransferase gene (*UFGT*), and anthocyanin 5-*O*-glucosyltransferase gene (*5GT*) showed consistent expression patterns, with their transcript levels being significantly higher in *V. heyneana* var. *adenoclada* than in CS and peaking during fruit maturation. Notably, these three genes showed higher expression in GH6 than in YN2. The glutathione S-transferase gene *GST4* (*Vaden04G010850*), which facilitates anthocyanin transport from the cytoplasm to the vacuole, showed significantly higher expression in *V. heyneana* var. *adenoclada* than in CS, with GH6 showing the highest expression level during late maturity among all tested samples.

### 3.5. Co-Expression Analysis of Phenolic Biosynthetic Genes and TFs

**Figure 6:**
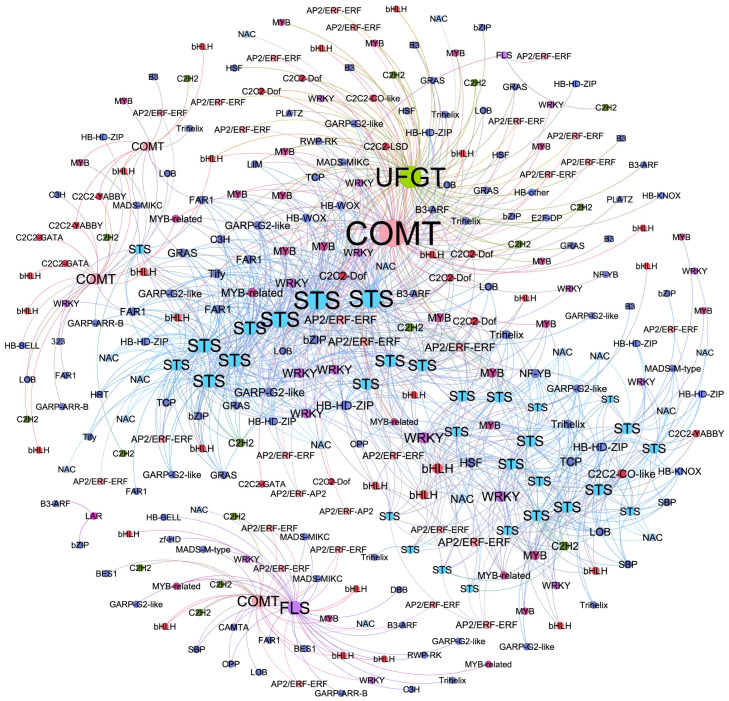
Co-expression analysis of genes encoding key enzymes and transcription factors involved in phenolic biosynthesis across three grape varieties. COMT, caffeic acid *O*-methyltransferase; STS, stilbene synthase; FLS, flavonol synthase; LAR, leucoanthocyanidin reductase; UFGT, UDP-glucose: flavonoid 3-*O*-glucosyltransferase.

TFs play a crucial role in plant growth, development, and stress responses. This study identified 1803 TFs spanning 96 gene families, including MYB (161), bHLH (129), ERF (128), C2H2 (112), FAR1 (97), and NAC (95), with MYB being the largest TF family (Table S5). To identify potential regulators of phenolic biosynthesis in *V. heyneana* var. *adenoclada*, we performed co-expression analysis between key structural genes involved in phenolic metabolism and all identified TFs. Using stringent screening criteria (|PCC| > 0.8, *p* < 0.05), we found that five key structural genes (*COMT*, *STS*, *FLS*, *LAR*, and *UFGT*) were strongly associated with 262 TFs. These TFs predominantly belonged to the ERF (29), MYB (27), bHLH (27), WRKY (17), NAC (16), C2C2 (16), and C2H2 (16) families, exhibiting either positive or negative regulatory relationships with the five structural genes (Figure 6).

Four members of *COMT* (*Vaden02G003300*, *Vaden16G023630*, *Vaden15G015010*, and *Vaden18G022230*), which encode key methyltransferases in phenolic acid biosynthesis, were positively regulated by the MYB, ERF, and WRKY families and negatively regulated by the bHLH, C2C2, and C2H2 families. Notably, we found that *COMT*s (*Vaden15G015010* and *Vaden16G023630*) were targeted by four activating transcription factors. These included two from the C2C2 family (*Vaden02G006430* and *Vaden06G013670*) and two from the bHLH family (*Vaden05G018820* and *Vaden12G001120*). A total of 141 TFs, primarily belonging to the MYB, ERF, bHLH, WRKY, and NAC families, regulated 31 *STS*s. Three *STS*s exhibited particularly high regulatory connectivity, functioning as hub genes: *Vaden16G017730* showed the highest number of connections (70 TFs), followed by *Vaden16G017900* (65 TFs) and *Vaden16G017840* (54 TFs). *FLS*, which encodes a key enzyme involved in flavonol biosynthesis, was represented by two members (*Vaden02G009600*, *Vaden18G003590*) that were regulated by 40 TFs mainly from the bHLH, ERF, MYB, and C2H2 families. Among these TFs, the majority were activators, with only 10 identified as repressors. *LAR* (*Vaden17G004570*), which encodes leucoanthocyanidin reductase involved in flavan-3-ol and proanthocyanidin biosynthesis, showed a remarkably simple regulatory pattern and was specifically activated by only two TFs, namely, B3-ARF (*Vaden04G010020*) and bZIP (*Vaden15G017290*). *UFGT* (*Vaden16G003070*), which encodes a key rate-limiting enzyme in anthocyanin synthesis, was involved in a complex network dominated by repressive regulation. Among the 61 connected TFs, 50 acted as negative regulators, whereas 11 functioned as activators. Among the 10 participating MYB TFs, only three (*Vaden02G015330*, *Vaden05G009210*, and *Vaden06G000710*) were identified as activators.

### 3.6. WGCNA

**Figure 7:**
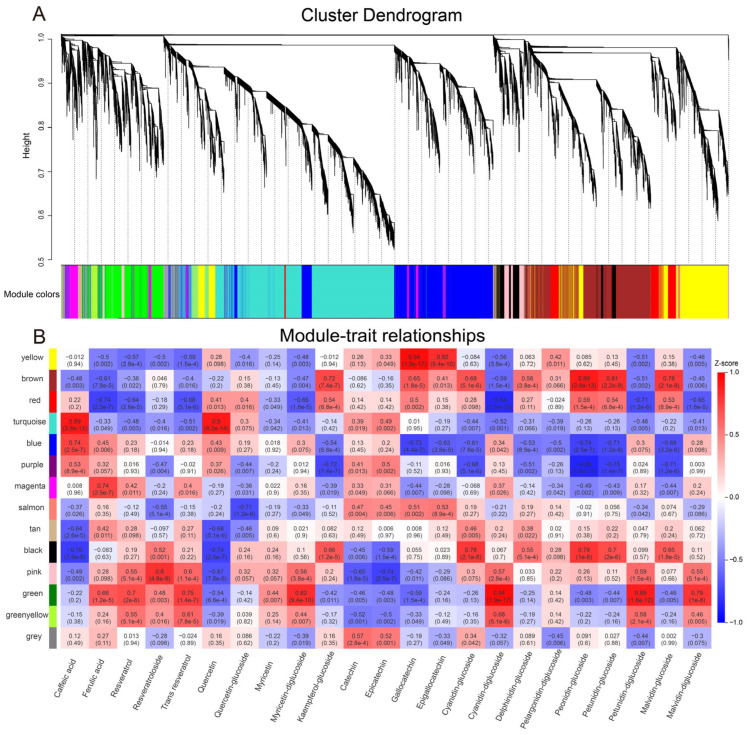
WGCNA-based co-expression module analysis: (**A**) cluster dendrogram showing co-expression modules identified through weighted gene co-expression network analysis (WGCNA) based on the topological overlap matrix, with major tree branches labeled with distinct color bands representing individual co-expression modules; (**B**) module–trait relationships illustrating the Pearson correlation coefficients and corresponding *p*-values (in parentheses) between module eigen-genes and specific phenolic metabolite traits. Each row corresponds to a color-coded module, and each column corresponds to a metabolite trait, with the color bar on the right indicating the correlation coefficient from low (blue) to high (red).

To elucidate the molecular mechanisms underlying metabolite accumulation and variation in *V. heyneana* var. *adenoclada*, we performed WGCNA to identify co-expression networks among the DEGs. Co-expression modules were defined as clusters of highly interconnected genes with strong correlation coefficients. The co-expression network is shown in Figure 7A, wherein genes with similar expression patterns were clustered into distinct modules through hierarchical clustering. The results revealed 13 gene clusters, each representing a unique module designated by a specific color. The turquoise module contained the largest number of genes (6961), followed by the blue module (4579).

Correlation analysis between the co-expression modules and specific phenolic compounds revealed several highly specific regulatory relationships (Figure 7B). The brown and black modules showed extremely significant positive correlations with major anthocyanin-3-*O*-glucosides, including peonidin-3-*O*-glucoside, delphinidin-3-*O*-glucoside, petunidin-3-*O*-glucoside, malvidin-3-*O*-glucoside, and cyanidin-3-*O*-glucoside. The turquoise module was significantly associated with the accumulation of both caffeic acid and quercetin, suggesting its potential role in regulating the biosynthesis of hydroxycinnamic acids and flavonols. Similarly, the magenta module showed a significant positive correlation with ferulic acid content, indicating its specific involvement in ferulic acid synthesis or metabolism. The pink module exhibited a highly significant positive correlation with resveratrol content, indicating that the module contains key regulatory genes for stilbene biosynthesis. Furthermore, the green and green-yellow modules showed significant positive correlations with various anthocyanin-3,5-*O*-diglucosides, including cyanidin-3,5-*O*-diglucoside, malvidin-3,5-*O*-diglucoside, and petunidin-3,5-*O*-diglucoside, suggesting their participation in the specific biosynthesis or transport of diglycosylated anthocyanins. KEGG enrichment analysis revealed that the DEGs within the identified modules were primarily involved in pathways related to metabolism, genetic information processing, environmental information processing, organismal systems, and cellular processes (Figure S4).

**Figure 8:**
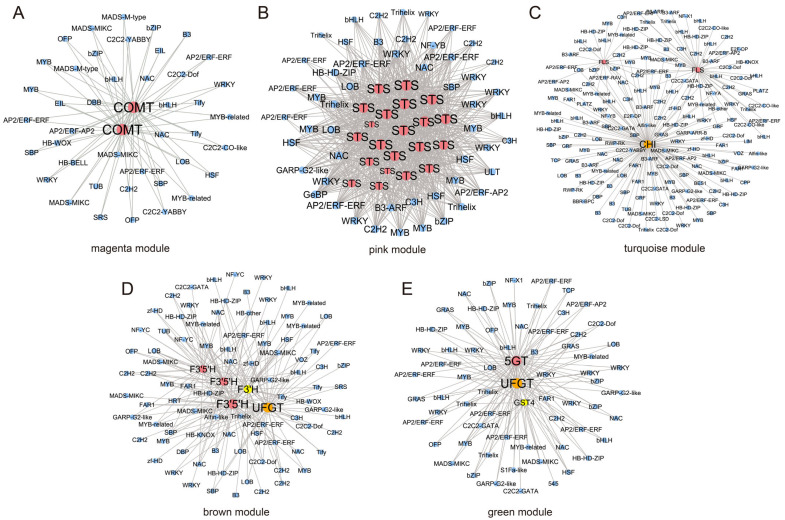
Co-expression networks linking phenolic biosynthetic structural genes with TFs across five WGCNA modules: (**A**) magenta module; (**B**) pink module; (**C**) turquoise module; (**D**) brown module; (**E**) green module. All nodes are shown as circles, with node size reflecting connectivity degree; larger nodes indicate higher connectivity. Blue nodes represent transcription factors, while red, yellow, and orange nodes represent structural genes.

The magenta module was associated with 42 TFs, with *YABBY2*, *LBD21*, *PIF4*, *SPL3*, *WRKY3*, and *MYB80* showing the highest connectivity (Table S6). Within this module, *COMT* (*Vaden16G023630* and *Vaden02G003330*) was identified, and TFs with correlation coefficients of >0.8 included *YABBY2* (*Vaden06G013670*), *bHLH9* (*Vaden12G001120*), *LBD21* (*Vaden13G000960*), *WRKY3* (Vaden01G015290), *SPL3* (*Vaden11G012750*), *MYB80* (*Vaden19G010780*), and *YABBY4* (*Vaden02G006430*) (Table S7). These TFs are likely involved in the regulation of ferulic acid biosynthesis (Figure 8A).

The pink module contained 61 TFs, predominantly from the AP2/ERF–ERF (10) and WRKY (9) families (Table S8). *STS*s were found within this module, and TFs with correlation coefficients of >0.8 included *WRKY75* (*Vaden01G021020*), *TGT3B* (*Vaden08G005440*), *WRKY72A* (*Vaden01G011760*), *MYB15* (*Vaden05G009210*), *MYB06* (*Vaden17G007270*), *MYB4* (*Vaden03G002220*), and *MYB36* (*Vaden11G004200*) (Table S9). These TFs are likely involved in resveratrol biosynthesis (Figure 8B).

The turquoise module was associated with 458 TFs, primarily from the MYB (59), bHLH (37), and HB (33) families (Table S10). This module contained key genes involved in flavonol biosynthesis, such as *CHI* (*Vaden19G000080*) and *FLS* (*Vaden13G000690* and *Vaden02G009550*). Expression regulation analysis indicated that *MYB5* (*Vaden08G020140*), *MYBS3* (*Vaden09G003920*), and *MYB61* (*Vaden07G006950*) positively regulated these genes (Table S11), suggesting their collaborative role in activating quercetin biosynthesis (Figure 8C).

The brown module contained 133 TFs, mainly from the MYB (15) and C2H2 (12) families, with *P2C27*, *NAC71*, *DOF36*, *ZAT11*, *MYB1*, and *NAC78* showing the highest connectivity (Table S12). Notably, *UFGT* (*Vaden11G017400*), *F3′H* (*Vaden17G008760*), and three *F3′5′H* isoforms (*Vaden02G006190*, *Vaden06G016370*, and *Vaden06G016330*) were present in this module. Further regulatory analysis (Figure 8D) revealed that the TFs *MYB1* (*Vaden02G015440*), *MYB44* (*Vaden18G008880* and *Vaden03G008050*), and *MYB113* (*Vaden02G015400*) positively regulated *F3′H*, *F3′5′H*, and *UFGT* expression, indicating their potential cooperative role in activating anthocyanin-3-*O*-glucoside biosynthesis (Table S13).

The green module was associated with 94 TFs, with *WRKY28*, *WRKY75*, *bHLH162*, *MYB1*, *ERF3*, and *NAC47* showing the highest connectivity (Table S14). Two key glycosyltransferase genes involved in anthocyanin biosynthesis, namely *UFGT* (*Vaden16G003070*) and *5GT* (*Vaden09G007990*), and *GST4* (*Vaden04G010850*), were significantly enriched in this module. Further regulatory network analysis revealed that multiple TFs showed high co-expression relationships (correlation coefficient > 0.8) with these three genes (Table S15). Specifically, *MYB1* (*Vaden02G015330*) and *MYB5* (*Vaden06G000710*) coordinately positively regulated all three genes (Figure 8E). These TFs may promote the biosynthesis, accumulation, and transport of anthocyanin-3,5-*O*-diglucosides through the synergistic regulation of *UFGT*, *5GT*, and *GST4* expression.

## 4. Discussion

As one of the origin centers of the genus *Vitis*, China possesses abundant wild grape resources, including *V. amurensis*, *V. davidii*, and *V. heyneana* [38]. Through long-term natural selection, these wild grapes have developed distinctive secondary metabolic characteristics, with their phenolic composition and content significantly differing from cultivated varieties [39,40,41,42,43,44]. For instance, the total phenolic content in the mature berry skins of *V. amurensis* is significantly higher than that of *V. vinifera* cultivars, such as Cabernet Sauvignon [45,46]. These phenolic compounds primarily include flavan-3-ols, flavonols (e.g., quercetin and kaempferol), phenolic acids (e.g., gallic acid and caffeic acid), and stilbenes (resveratrol and its derivatives). Notably, certain wild *Vitis* species and their interspecific hybrid offspring exhibit a remarkable capacity for resveratrol accumulation, with the resveratrol content in the berry skin of wild-sourced rootstocks reaching up to 50 times higher than that of cultivated varieties [47]. Furthermore, the resveratrol content in Chinese wild grapes is generally higher than that in cultivars [48].

Consistent with these findings, our integrated metabolomic and transcriptomic analysis revealed that *V. heyneana* var. *adenoclada* grapes exhibit significant accumulation of multiple phenolic compounds compared to *V. vinifera* grapes. The integration of transcriptomics and metabolomics has emerged as a powerful strategy for deciphering complex metabolic networks in non-model plant species [49]. This approach enables simultaneous assessment of transcriptional changes and their metabolic outcomes, facilitating the identification of key regulatory nodes controlling metabolite accumulation [24]. Multi-omics integration has been shown to accelerate the discovery of bioactive compounds and reveal mechanisms undetectable by single-omics approaches [24,28]. Moreover, this strategy has been successfully applied to dissect spatiotemporal metabolic dynamics and construct regulatory networks in various plant species [49,50]. By applying this integrated framework, we were able to link the elevated expression of multiple biosynthetic genes—from *PAL* and *C4H* in the early phenylpropanoid pathway to *UFGT* and *GST4* in anthocyanin synthesis and transport—with the enhanced accumulation of phenolic acids, stilbenes, flavonols, and anthocyanins in *V. heyneana* var. *adenoclada* (Figure 5).

This study revealed that *V. heyneana* var. *adenoclada* exhibits significant accumulation of multiple phenolic compounds. Specifically, the contents of phenolic acids (caffeic and ferulic acids), resveratrol, flavonols (quercetin and myricetin), flavan-3-ols (catechin and epicatechin), and anthocyanin-3,5-*O*-diglucosides were significantly higher in *V. heyneana* var. *adenoclada* than in CS. Notable genotypic differences in phenolic composition are observed among germplasms of wild grape varieties. For example, *V. yanshanensis* is characterized by high anthocyanin accumulation, *V. davidii* shows a high total flavan-3-ol content, and *V. heyneana* and *V. adenoclada* exhibit high levels across multiple phenolic metrics, including the total phenolic and total flavonoid contents [51]. Within *V. heyneana* var. *adenoclada*, GH6 contained significantly higher levels of ferulic acid, resveratrol, myricetin, and anthocyanin-3,5-*O*-diglucosides than YN2 at maturity, whereas YN2 showed greater accumulation of quercetin, catechin, and epicatechin than GH6 at the green berry stage (Figure 3B). These results indicate that GH6 favors stilbene and anthocyanin diglucoside accumulation during maturation, whereas YN2 prioritizes flavan-3-ol biosynthesis during early development, revealing significant spatiotemporal regulatory differences in phenolic metabolism between the two varieties. Such differences not only reflect metabolic genetic diversity but also provide a theoretical foundation for breeding new grape varieties with enhanced phenolic content and stress resistance.

*C4H* and *COMT* encode key enzymes that play sequential and crucial roles in phenolic acid biosynthesis [52,53,54,55,56]. The enzyme encoded by *C4H* catalyzes the conversion of cinnamic acid to p-coumaric acid, acting as an early rate-limiting enzyme that directs carbon flux in the pathway [57], whereas the *COMT*-encoded enzyme mediates the methylation of caffeic acid to ferulic acid [58,59]. Herein, the significantly higher expression of *C4H* and *COMT* in *V. heyneana* var. *adenoclada* compared to that in CS was consistent with the higher accumulation of caffeic and ferulic acids observed in metabolomic analysis (Figure 3B and Figure 5), suggesting that increased expression of these genes is responsible for enhanced phenolic acid biosynthesis in *V. heyneana* var. *adenoclada*. *STS*, which encodes a key rate-limiting enzyme in stilbene biosynthesis with antimicrobial and stress-resistant properties, contributes to plant defense mechanisms under stress conditions [60,61,62,63,64]. Herein, the marked upregulation of *STS* expression at fruit maturity corresponded to the significant increase in resveratrol content in *V. heyneana* var. *adenoclada* (Figure 3B), indicating that transcriptional activation of *STS* is a crucial regulatory factor driving stilbene accumulation. The expression levels of *CHI* and *FLS* were notably higher in *V. heyneana* var. *adenoclada*, indicating overall enhancement of the flavonol synthesis pathway. Notably, *F3′5′H* exhibited genotype-specific high expression during fruit maturation, with significantly higher transcript levels in GH6 than in YN2 (Figure 5). This expression pattern is consistent with the metabolomic data showing higher accumulation of myricetin, delphinidin, petunidin, and malvidin derivatives in *V. heyneana* var. *adenoclada* at maturity, particularly in GH6 (Figure 3B). These results suggest that differential expression of *F3′5′H* is a key regulatory node governing the variation in myricetin and tri-hydroxylated anthocyanin contents between cultivars. These findings highlight the stronger metabolic potential of *V. heyneana* var. *adenoclada*, especially the GH6 genotype, in B-ring hydroxylation of flavonoids. *LDOX*, which encodes a key catalyst in anthocyanin biosynthesis, converts leucoanthocyanidins to colored anthocyanidins, serving as a critical junction connecting upstream flavonoid synthesis with downstream pigment modification [65,66,67,68,69]. The significantly higher expression of *LDOX* in *V. heyneana* var. *adenoclada* than in CS, accompanied by the coordinated upregulation of *UFGT*, *5GT*, and *GST4* during maturation, particularly in GH6, collectively facilitates the efficient synthesis and transport of anthocyanin-3,5-*O*-diglucosides (Figure 3B and Figure 5). Chinese wild grapes predominantly accumulate diglycosylated anthocyanins in the berry skin, contrasting sharply with the accumulation of monoglycosylated anthocyanins typically observed in *V. vinifera* [43].

To elucidate the regulatory networks underlying phenolic metabolism in different grape cultivars, we performed co-expression analysis on structural genes and TFs. Existing studies have primarily focused on the transcriptional regulation of phenolic metabolism in *V. vinifera* and related cultivated grape species [70,71,72,73,74], whereas the regulatory networks in East Asian wild grape species remain largely unclear. Herein, we identified 1803 TFs, with the MYB family being the most abundant (Figure 6, Table S5). In grapes, *MYB12* (*MYBF1*) has been identified as a specific regulator of flavonol synthesis, directly activating the promoters of *FLS* and *UFGT* and showing light-induced expression patterns consistent with flavonol accumulation [71]. We found that *MYB12* (*Vaden07G005380*) positively regulated *COMT* (*Vaden15G015010*), *CHI* (*Vaden13G002060*), *DFR* (*Vaden18G012180*), and *LAR* (*Vaden17G004570*) (Table S16). Furthermore, WGCNA revealed that *MYB5* (*Vaden08G020140*), *MYBS3* (*Vaden09G003920*), and *MYB61* (*Vaden07G006950*) expression showed a significant positive correlation with *FLS* (*Vaden13G000690* and *Vaden02G009550*) expression (Figure 8C), suggesting that these three TFs activate *FLS* expression and positively regulate quercetin biosynthesis in *V. heyneana* var. *adenoclada*.

Stilbene biosynthesis is primarily regulated by the synergistic action of the WRKY (VqWRKY53), MYB (VqMYB14 and VqMYB15), and AP2/ERF (VqERF062 and VqERF1B) families [75,76,77,78]. These TFs form complex protein interaction networks (VqWRKY53 with VqMYB14/MYB15 and VqERF062 with VqERF1B) that recognize and bind to the promoter regions of *STS*, thereby regulating *STS* expression and promoting stilbene biosynthesis to enhance disease resistance [12,79,80]. Herein, we identified multiple MYB family TFs that positively regulate *STS* expression (Figure 6). In addition to *MYB15* (*Vaden05G009210*), which is a known regulator of *STS*, *MYB06* (*Vaden17G007270*), *MYB4* (*Vaden03G002220*), and *MYB36* (*Vaden11G004200*) significantly enhanced the transcriptional activation of *STS*, suggesting their involvement in resveratrol biosynthesis during grape maturation (Figure 8B).

In grapes, MYBA1 not only directly activates the transcription of *UFGT* and *DFR* but also shows a significant positive correlation with anthocyanin content [81,82,83,84,85]. We found that *MYB1* (*Vaden02G015330*) and *MYB5* (*Vaden06G000710*) positively regulated key genes involved in anthocyanin synthesis and transport (*UFGT*, *5GT*, and *GST4*) (Figure 8C). Through coordinated upregulation of these genes, MYB1 and MYB5 jointly promote the efficient synthesis, accumulation, and vacuolar transport of anthocyanin-3,5-*O*-diglucosides in *V. heyneana* var. *adenoclada*, thereby explaining the differential accumulation of monoglycosylated and diglycosylated anthocyanins between this wild grape variety and CS. *5GT* is a key enzyme catalyzing the formation of anthocyanin-3,5-*O*-diglucosides [86,87], and diglycosylated anthocyanins represent an important metabolic characteristic of Chinese wild grapes [43,88].

## 5. Conclusions

This study aimed to identify key phenolic compounds and their dynamic changes, determine key structural genes and regulators involved in phenolic metabolism, and construct molecular regulatory networks underlying phenolic biosynthesis in *V. heyneana* var. *adenoclada* through integrated metabolomic and transcriptomic analyses.

At the metabolic level, *V. heyneana* var. *adenoclada* exhibited significantly higher levels of phenolic acids (caffeic and ferulic acids), stilbenes (resveratrol), flavonols (quercetin and myricetin), flavan-3-ols (catechin and epicatechin), and anthocyanin-3,5-*O*-diglucosides than *V. vinifera*. Furthermore, genotype-specific accumulation patterns were observed in *V. heyneana* var. *adenoclada*: GH6 accumulated higher levels of ferulic acid, resveratrol, myricetin, and anthocyanin diglucosides than YN2 at maturity, whereas YN2 showed greater accumulation of quercetin and flavan-3-ols at the green berry stage.

Transcriptional regulation analysis indicated that multiple MYB TFs formed a sophisticated regulatory network that coordinately controlled the expression of key structural genes involved in phenolic metabolism. *MYB12* (*MYBF1*) expression showed significant positive correlations with the expression of several methyltransferase and reductase genes (*COMT*, *CHI*, *DFR*, and *LAR*), suggesting its role as a master upstream regulator involved in the coordinated activation of polyphenol synthesis. *MYB5*, *MYBS3*, and *MYB61* promoted quercetin synthesis by positively regulating *FLS* expression. *MYB06*, *MYB4*, and *MYB36* enhanced *STS* transcription to drive resveratrol biosynthesis. MYB1 and *MYB5* synergistically activated the expression of *UFGT*, *5GT*, and *GST4*, thereby facilitating the efficient synthesis and transport of anthocyanin diglucosides.

The high accumulation of phenolic compounds in *V. heyneana* var. *adenoclada* results from the marked upregulation of multiple structural genes and relies on precise coordination by a complex regulatory network composed of MYB TFs. However, the observed temporal differences between gene expression and end-product accumulation suggest that, in addition to transcriptional regulation, post-translational modifications, substrate availability, and metabolic flux distribution collectively regulate the final efficiency of phenolic biosynthesis. These findings provide an important theoretical foundation and introduce candidate genes for further investigation on the regulatory mechanisms underlying phenolic biosynthesis in grapes and for improving the quality of grape varieties and their wines.

## Figures and Tables

**Table 1:** Physicochemical parameters of grapes from three varieties at harvest.

Cultivar/Variety	GH6	YN2	CS
Berry fresh weight (g)	1.62 _a_ ± 0.14	1.72 _a_ ± 0.10	1.58 _a_ ± 0.08
Soluble solid content (°Brix)	11.33 _b_ ± 0.95	9.07 _c_ ± 0.60	16.93 _a_ ± 0.51
pH	3.21 _b_ ± 0.07	3.15 _b_ ± 0.02	3.68 _a_ ± 0.01
Titratable acidity (g/L)	16.90 _a_ ± 1.60	17.23 _a_ ± 0.98	6.63 _b_ ± 0.20

Note: Data are presented as mean ± SD (*n* = 3). Different letters within the same row indicate significant differences among treatments at *p* < 0.05, as determined by one-way ANOVA followed by Duncan’s new multiple range test.

## Data Availability

The RNA-seq raw data have been uploaded to NGDC (https://ngdc.cncb.ac.cn/), BioProject Accession: PRJCA024858. The other data used for the analysis in this study are available within the article and the Supplementary Materials. Further inquiries can be directed to the corresponding authors.

## Supplementary Materials

The following supporting information can be downloaded at https://www.mdpi.com/article/10.3390/foods15142574/s1: Figure S1: KEGG enrichment analysis of differential metabolites in (A) GH6-EL38 and CS-EL38 and (B) YN2-EL38 and CS-EL38; Figure S2: K-means cluster analysis of metabolite profiles in three grape varieties; Figure S3: Classification and statistics of metabolites in seven subclasses; Figure S4: KEGG enrichment analysis of DEGs in six modules: (A) brown module, (B) turquoise module, (C) black module, (D) pink module, (E) green module, and (F) green-yellow module; Table S1: Metabolite content of different grapes; Table S2: Metabolite content in seven subclasses; Table S3: RNA-seq statistics of 36 grape samples; Table S4: All identified DEGs in 36 samples from three grapes; Table S5: Transcription factor prediction; Table S6: Genes of magenta module; Table S7: The correlation coefficient of *COMT* and transcription factor in magenta module; Table S8: Genes of pink module; Table S9: The correlation coefficient of *STS* and transcription factor in pink module; Table S10: Genes of turquoise module; Table S11: The correlation coefficient of *FLS*, *CHI* and transcription factor in turquoise module; Table S12: Genes of brown module; Table S13: The correlation coefficient of *UFGT*, *F3′H*, *F3′5′H* and transcription factor in brown module; Table S14: Genes of green module; Table S15: The correlation coefficient of *UFGT*, *5GT*, *GST4* and transcription factor in green module; Table S16: The correlation coefficient of MYB12 transcription factor and phenolic structural genes.
