# Supplementary material for: Integrated Metabolomics and Transcriptomics Provided Novel Insights into the Biosynthetic Regulation of Phenolic Compounds in Vitis heyneana Roem. et Schult. var. adenoclada (Hand.-Mazz.)
Source: Foods. 2026 Jul 22;15(14):2574. doi: 10.3390/foods15142574 (PMC13408528; doi:10.3390/foods15142574)
Supplement: Supplementary file 1 [file foods-15-02574-s001.zip › Supplementary Figures.pdf]

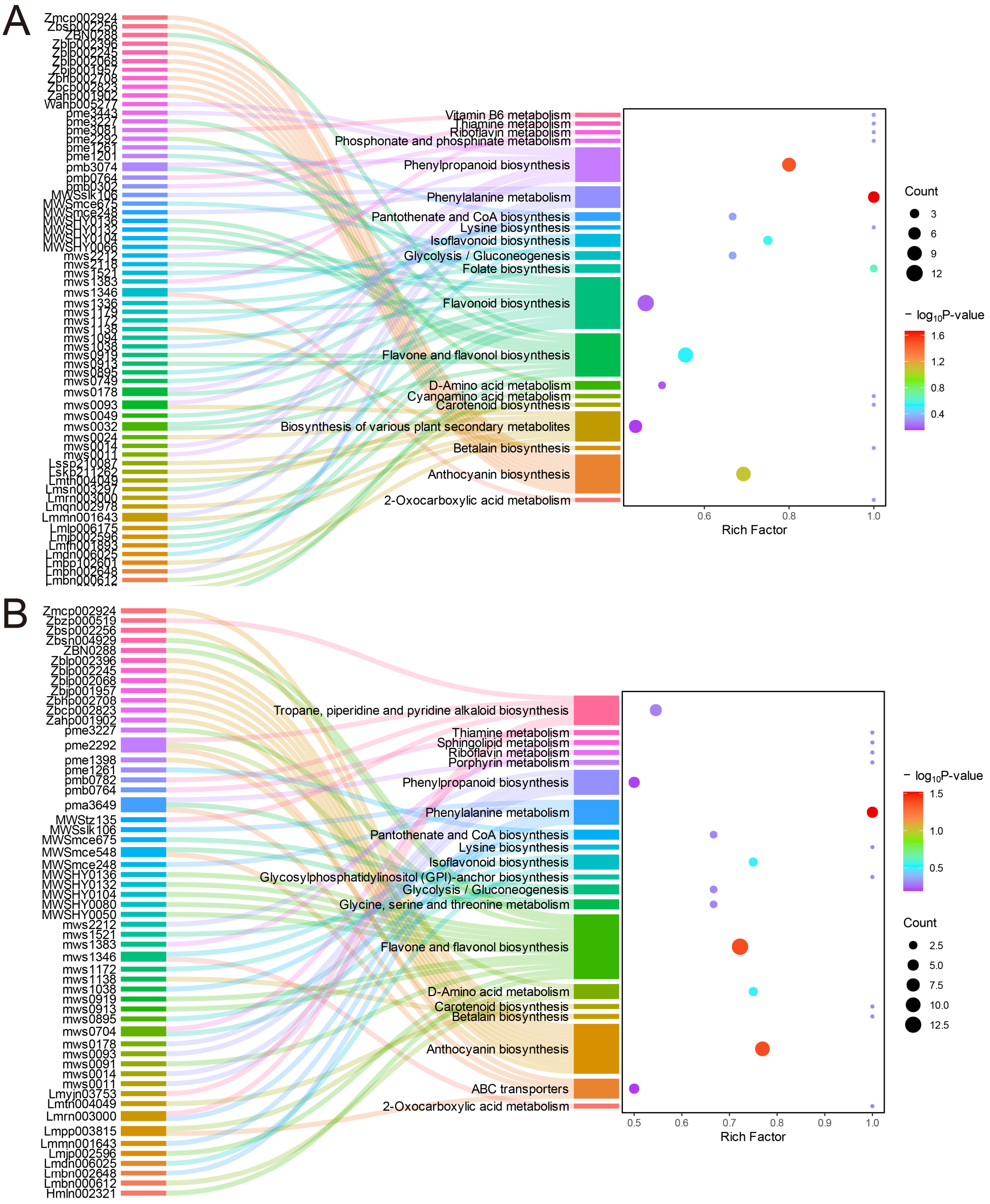

Figure S1. KEGG enrichment analysis of differential metabolites in (**A**) GH6-EL38 and CS-EL38 and (**B**) YN2-EL38 and CS-EL38.

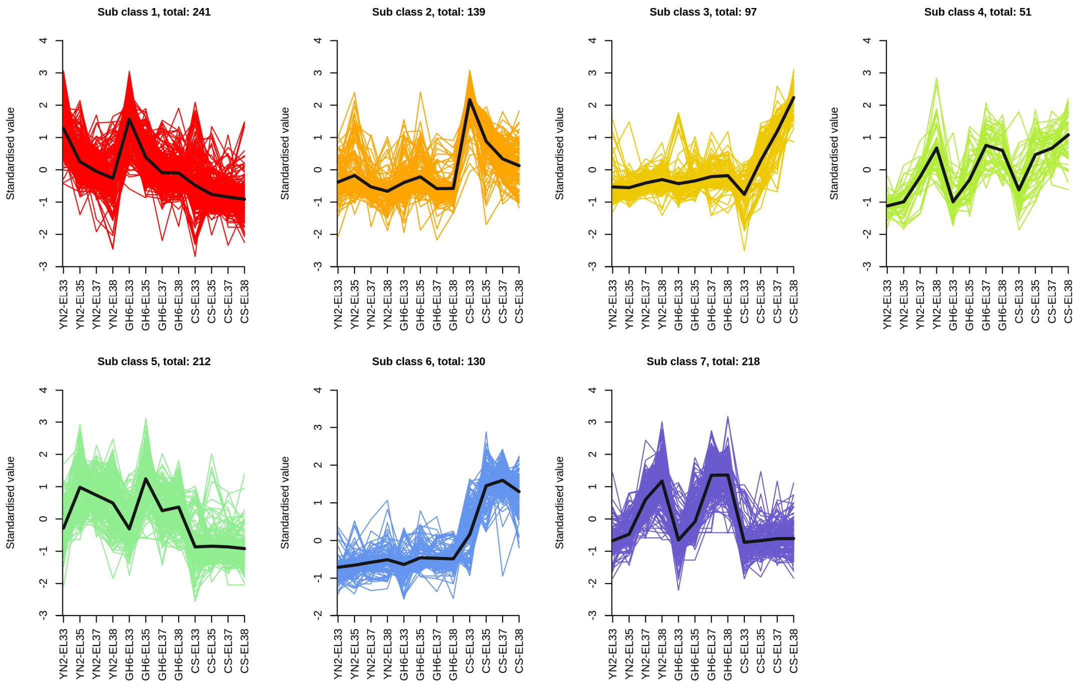

Figure S2. K-means cluster analysis of metabolite profiles in three grape varieties.

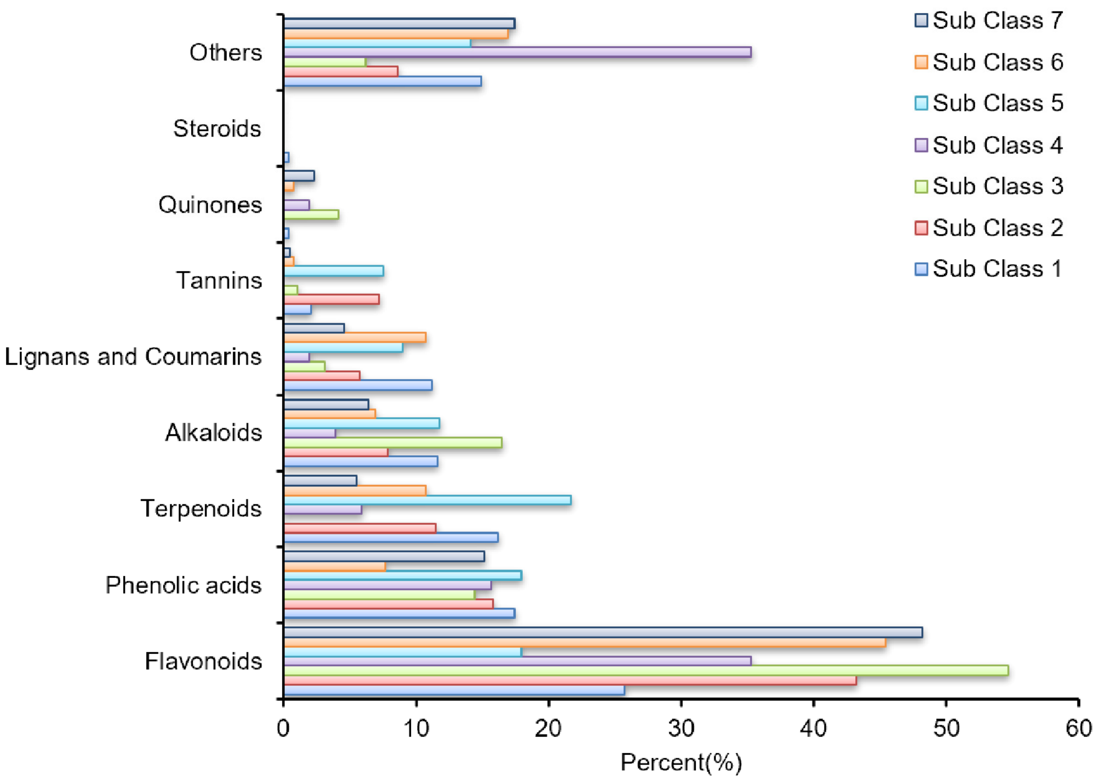

Figure S3. Classification and statistics of metabolites in seven subclasses.

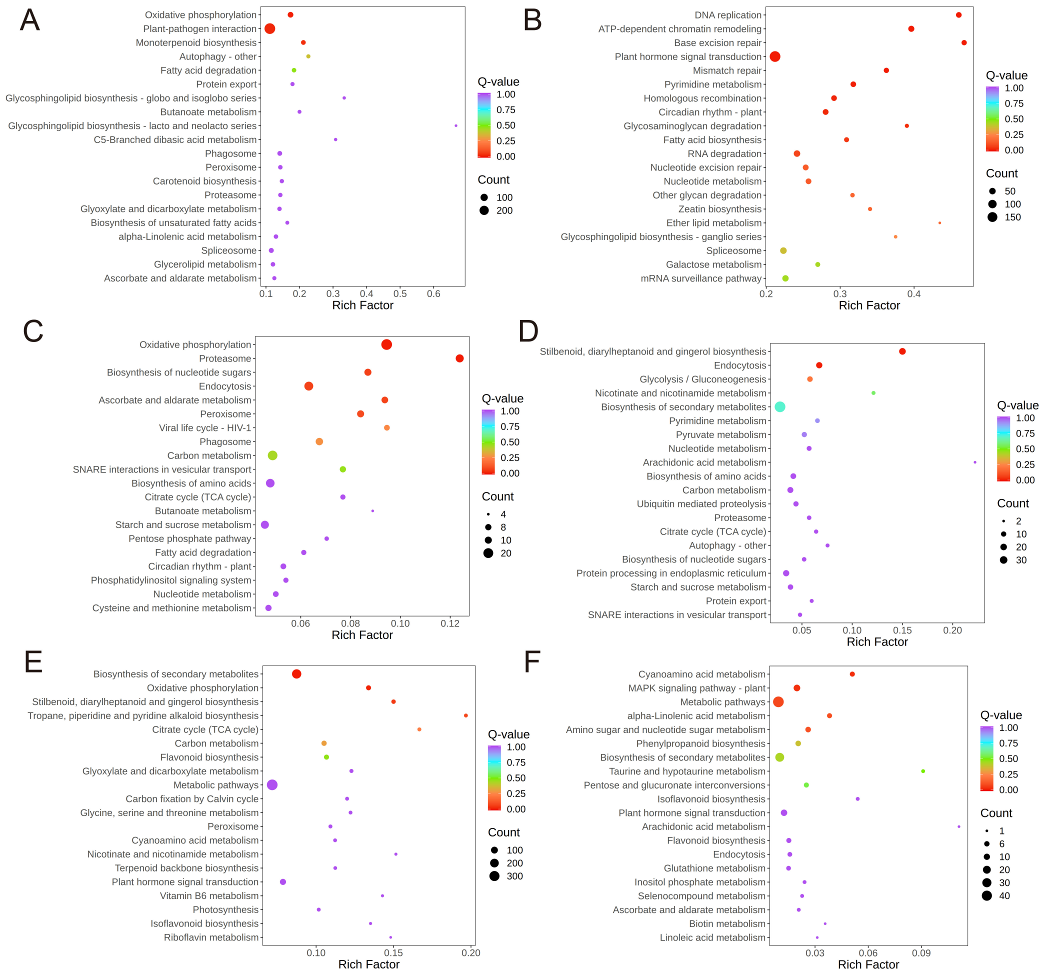

Figure S4. KEGG enrichment analysis of DEGs in six modules: (**A**) brown module, (**B**) turquoise module, (**C**) black module, (**D**) pink module, (**E**) green module, and (**F**) green-yellow module.
